# Inhibiting the CD8^+^ T cell infiltration in the tumor microenvironment after radiotherapy is an important mechanism of radioresistance

**DOI:** 10.1038/s41598-018-30417-6

**Published:** 2018-08-09

**Authors:** Hai-yan Chen, Lei Xu, Lin-feng Li, Xiao-xing Liu, Jian-xin Gao, Yong-rui Bai

**Affiliations:** 1 Department of radiation oncology, Renji Hospital, School of Medicine, Shanghai Jiaotong University, Shanghai Shi, China; 2 State Key Laboratory of Oncogenes and Related Genes, Renji-Med X Clinical Stem Cell Research Center, Renji Hospital, School of Medicine, Shanghai Jiaotong University, Shanghai Shi, China

## Abstract

Endogenous immune response participates in tumor control, and radiotherapy has immune modulatory capacity, but the role of immune modulation in the tumor microenvironment invoked by radiotherapy in radiosensitivity is poorly defined. In the present study, a radio-resistant melanoma cell line was obtained after repeated irradiation to the parental tumor in C57BL/6 mice. Radiotherapy resulted in aggregation of CD8^+^ and CD3^+^ T cells, and decrease of myeloid-derived suppressor cells and dendritic cells in the parental tumor, but not in the resistant tumors. CD4^+^ T cells and B cells did not change significantly. The CD8^+^ T cell infiltration after radiotherapy is important for tumor response, because in the nude mice and CD8^+^ T cell-depleted C57BL/6 mice, the parental and resistant tumor has similar radiosensitivity. Patients with good radiation response had more CD8^+^ T cells aggregation after radiotherapy. Radiotherapy resulted in robust transcription of T cell chemoattractant in the parental cells, and the expression of CCL5 was much higher. These results reveal a novel mechanism of radioresistance, tumor cells inhibit the infiltration of CD8^+^ T cell after radiotherapy and become radioresistant. Increasing CD8^+^ T cell infiltration after RT may be an effective way to improve tumor radiosensitivity.

## Introduction

Radiation therapy (RT) has been used for over one hundred years to treat patients with cancer, but the local control is still poor in some patients. To improve the efficacy of radiotherapy, it is important to understand the mechanisms of radioresistance. Previously inherent cellular radiosensitivity is hypothesized to account for this discrepancy^1,2^. In recent decades, with the development of immunology, the participation of endogenous immune system in modifying radiation effect has been widely documented^3–5^. Radiotherapy has immune modulatory capacities^6–10^. Following irradiation, tumor cells express more MHC-II, release a large amount of tumor associated antigens and other molecules, these enable antigen-presenting cells to stimulate a tumor-specific immune response. T cells accumulate after ablative radiotherapy, and depletion of CD8^+^ T cells significantly impairs radiation effect^3–5,11,12^. Radiation also induce a rapid and transient infiltration of neutrophils into tumors^13^. Recruitment of myeloid-derived suppressor cells (MDSC) after RT, on the opposite, regulates radiation response by suppressing T cell function and exerts immunosuppressive effect in the tumor microenvironment (TME)^14^.

It is well known that some tumors are more radiosensitive than the others, but the role of immune responses in such different radiosensitivity is poorly defined. Given the participation of endogenous immune responses in tumor control, we investigated whether tumors with different radiosensitivity *in vivo* had different immune activation after radiotherapy, and whether this had functional consequences.

## Results

### The radioresistant tumor cell has radiosensitivity similar to the parental cell *in vitro*

**Figure 1:**
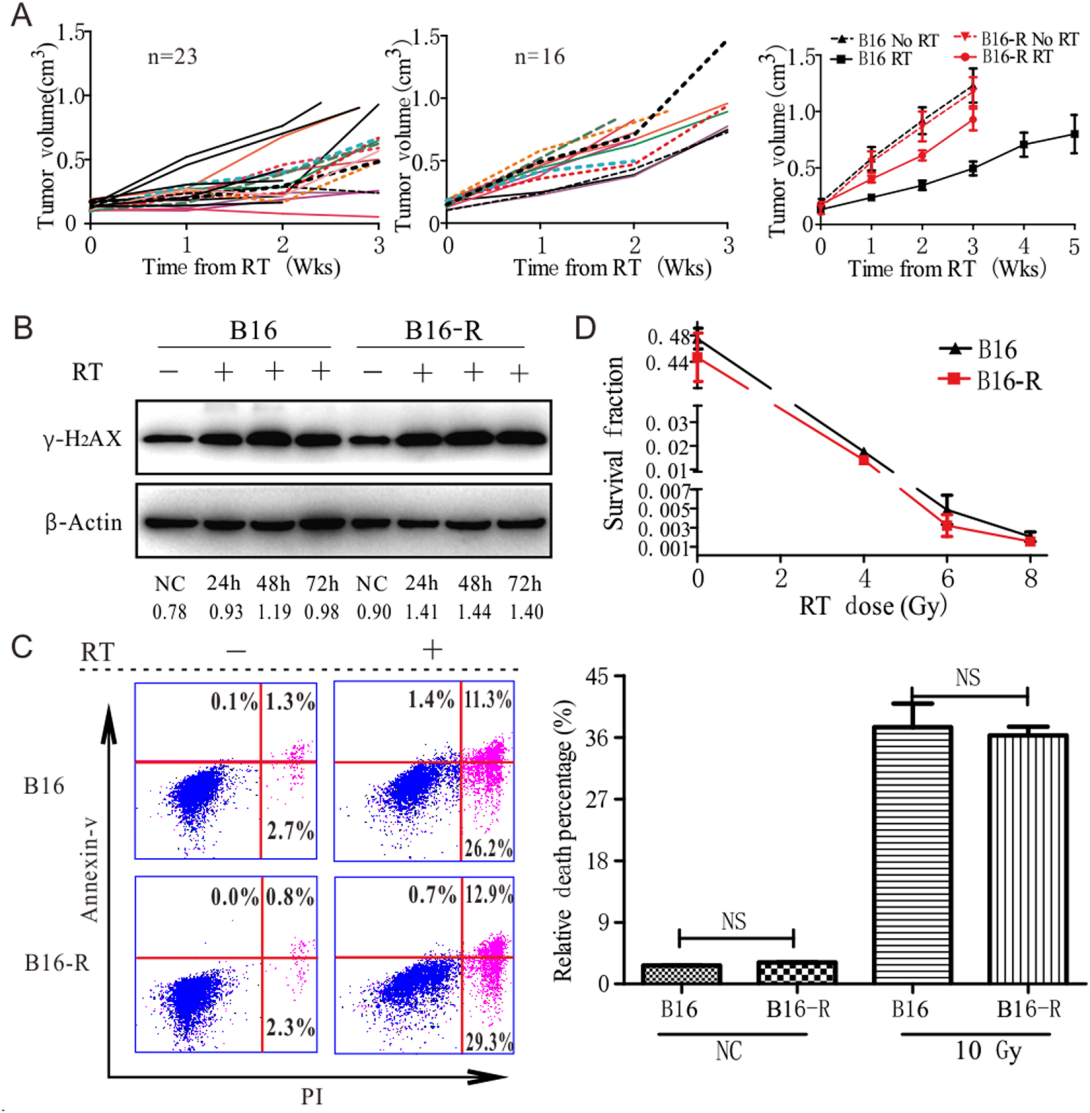
The radioresistant and parental tumor have different radiosensitivity not associated with classic factors. (**A**) Subcutaneous inoculation revealed that B16-R tumors were radioresistant in C57BL/6 mice while untreated tumors have a similar growth rate, data points were represented as mean ± SEM. (**B**) The expression of γ-H2AX increased after radiotherapy, and was similar between B16 and B16-R. Death analysis by FACS. (**C**) shown that they had similar death rate 48 hours after 10 Gy. (**D**) Clonogenic survival to evaluate intrinsic factors of radioresistance in culture showed no significant differences between the two tumor clones, data points were mean ± SD.

Local irradiation was given to the melanoma in C57BL/6 mice, tumor with poor radiation response was resected, cultured and passaged *in vitro*. Inoculation of these tumor cells and irradiation was repeated until a radioreistant cell line was obtained. Compared to the parental tumors, the regrowth rate of the radio-resistant tumor after radiotherapy was much faster (Fig. 1A). To address the potential contribution of inherent cellular radiosensitivity in the tumor radiation response, *in-vitro* experiments were used. Radiation-induced γ H2AX foci in the nucleus is routinely used to access the amount of DNA damage and repair kinetics, so we checked the expression of γ H2AX, it increased after 10 Gy in both cell lines, and found that the expression was not less in the resistant cell (Fig. 1B, full-length unedited blots/gels are presented in Fig. S1). Apoptosis and necrosis evaluation after 10 Gy (Fig. 1C) shown that similar percentage of cells died at the acute phase (48 h after RT), also there was no significant difference in clonogenicity (Fig. 1D). These results suggested that autonomous factors were not responsible for the different regrowth kinetics *in vivo* after RT, and the host factors may contribute to this difference.

### CD8^+^ T cell infiltration is different in the parental and resistant tumor after radiotherapy

In order to figure out the possible contribution of immune response in tumor radiosensitivity, tumors were given 30 Gy and harvested on the 14^th^ day to analyze the tumor infiltrating leucocytes (TILs).

**Figure 2:**
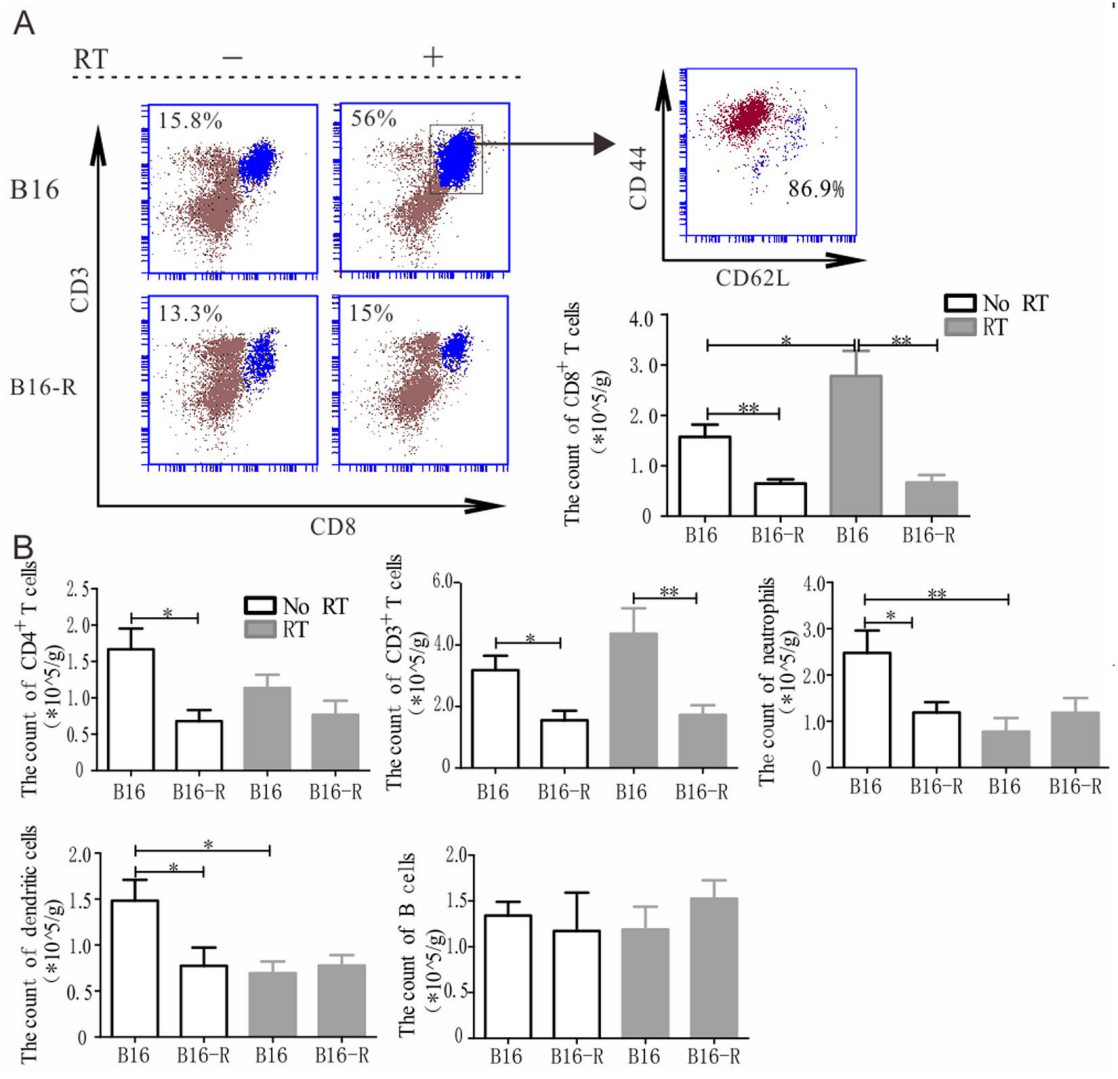
Radiotherapy is associated with a differential antitumor immune response. (**A**) There is an influx of CD8^+^ T cells 14 days from radiotherapy in the parental tumor, but with fewer change in the resistant tumor. Most of the CD8^+^ T cells are CD44^+^CD62L^−^ (gated at CD3^+^CD8^+^ cells). (**B**) There was no significant difference in the density of other immune cells in the tumor microenvironment between the parental and resistant tumor after radiotherapy except CD3^+^ T cells. Data points were data ± SEM.

FACS of CD3 and CD8 revealed substantial number of CD8^+^ T cell in the untreated parental tumors that increased after radiotherapy (Fig. 2A), most of which were effector T cell (CD44^+^CD62L^−^); in contrast, there were few CD8^+^ T cells with or without RT in the resistant tumors. The percentage of CD8^+^ T cell in TILs did not differ significantly in the parental and resistant tumor without RT, total TILs were less in the resistant tumors, and there was more infiltrated CD8^+^ T cell in the parental tumor. Due to the low CD8^+^ T cell percentage and TIL count, the density of CD8^+^ T cell was lower in the resistant tumors after RT compared to the parental tumors (P < 0.01 by Mann-Whitney U test, Fig. 2B). Meanwhile total CD3^+^ T cell was higher in the parental tumors no matter before or after radiotherapy (P < 0.01 by Mann-Whitney U test, Fig. 2B), though the change of CD4^+^ T cell was not so obvious.

MDSC (CD11b^+^Gr1^+^) was abundant in the parental tumor at baseline, and there was a dramatic decrease after radiotherapy; but the change was small in the resistant tumor. MDSC were further classified based on the expression of Ly6C and Ly6G as previously described^14^, and we found that the percentage of CD11b^+^ Ly6G^+^ decreased after radiotherapy in the parental tumor, with an increase of CD11b^−^ Ly6C^+^ population, and the CD11b^−^ Ly6C^+^ population was much higher in the parental tumor than the resistant tumor after radiotherapy (Fig. S2).

More dendritic cells (CD11c^+^ MHCII^+^) infiltrated in the parental tumor at baseline and decreased after radiotherapy. In contrast, there was little change in the radio-resistant tumors, and the density was similar in the parental and resistant tumors after treatment (Fig. 2B). The infiltration of B cell (Fig. 2B) did not change a lot after RT, and was comparable in the parental and resistant tumors no matter radiation or not.

Taken together, radiation reprogrammed the TME in the parental tumor, but did not brought great change to the resistant tumor, and the upmost difference was the much more infiltration of CD8^+^ T cell in the parental tumor. This raised the possibility that CD8^+^ T cell infiltration post radiotherapy might related to tumor radiosensitivity *in vivo*.

### Patients with good radiation response had more CD8^+^ T cell infiltration in the TME

**Figure 3:**
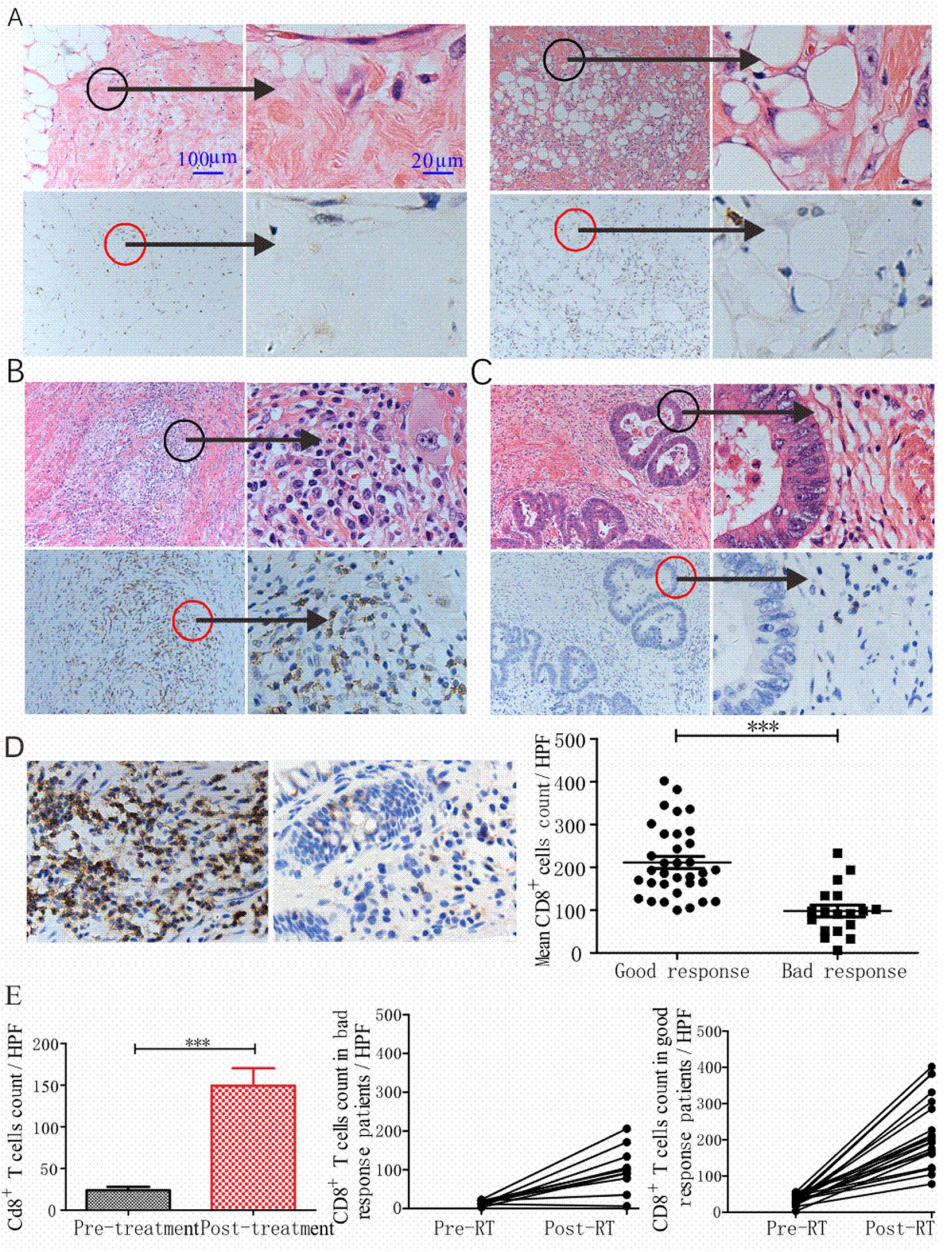
Histologic patterns of CD8^+^ T cell in human tumors and link to response to RT. (**A**) A tumor foci in the muscularis of the rectum, which was replaced by fibrotic stroma and porosis after RT. The left represented the tumor stromal area, and the right represented the intratumoral area. Low magnification ×4, high magnification ×40. (**B**) A tumor foci inside muscularis post RT, most of the tumor cells disappeared, a characteristic tumor cell with multilobated nulear and expanded cytoplasm was left. Abundant CD8^+^ T cells infiltrated. (**C**) A tumor foci with most of the tumor cells alive after RT, and little CD8^+^ T cells infiltrated. (**D**) Representative images of high and low infiltration of CD8^+^ T cells (×40), in the 52 patients, tumors with good response to RT had more infiltration than the bad response group. (**E**) In the 32 pairs of pre- and post RT specimens, more CD8^+^ T cells infiltrated after radiotherapy, and patients with bad response had less CD8^+^ cells both before and after RT.

Since there was great difference in the infiltration of CD8^+^ T cell after radiotherapy in the murine tumors with different radiosensitivity, we verified this phenomenon in patients with rectum cancer who received neoadjuvant chemoradiotherapy. Tumor response in the surgery specimen was evaluated using the tumor regression grade (TRG) system as described^15,16^. Among them 18 patients (34.6%) were graded as TRG 1–2, classified as “bad response”, the remaining 34 patients (65.4%) were good response graded as TRG 3, no TRG 4 was observed. On the slides, some of the tumor tissues were replaced by fibrotic stroma or porosis, with no tumor cells in the primary tumor bed; under such condition, the infiltration of CD8^+^ T cells was little (Fig. 3A). In some areas most of the tumor cells disappeared, left several tumor cells with very large nuclei often bizarrely shaped and multilobated, and the cytoplasm in the tumor cells expanded and had a dense amphophilic quality with disappearance of cell borders and sometimes vacuolation. Under such condition, a great deal of CD8^+^ T cells infiltration usually could be seen (Fig. 3B). The third condition is that most of the tumor cells survived without obvious injury, and little CD8^+^ T cells infiltrated here (Fig. 3C).

The infiltration of CD8^+^ T cell was analyzed in the good and bad response groups, and found that there was significant difference (P = 0.0022), with more CD8^+^ T cells in the good response group (Fig. 3D). One way anova analysis showed that the response type (good or bad response) closely related to the cell count, with P = 0.015. Compared the pre-treatment specimens with post-treatment ones, CD8^+^ cells aggregated significantly after treatment (P < 0.001). Patients with bad response had less CD8^+^ T cells both before and after treatment (Fig. 3E).

### The difference of radiosensitivity between the parental and resistant cells shrank in T cell-deficient mice

**Figure 4:**
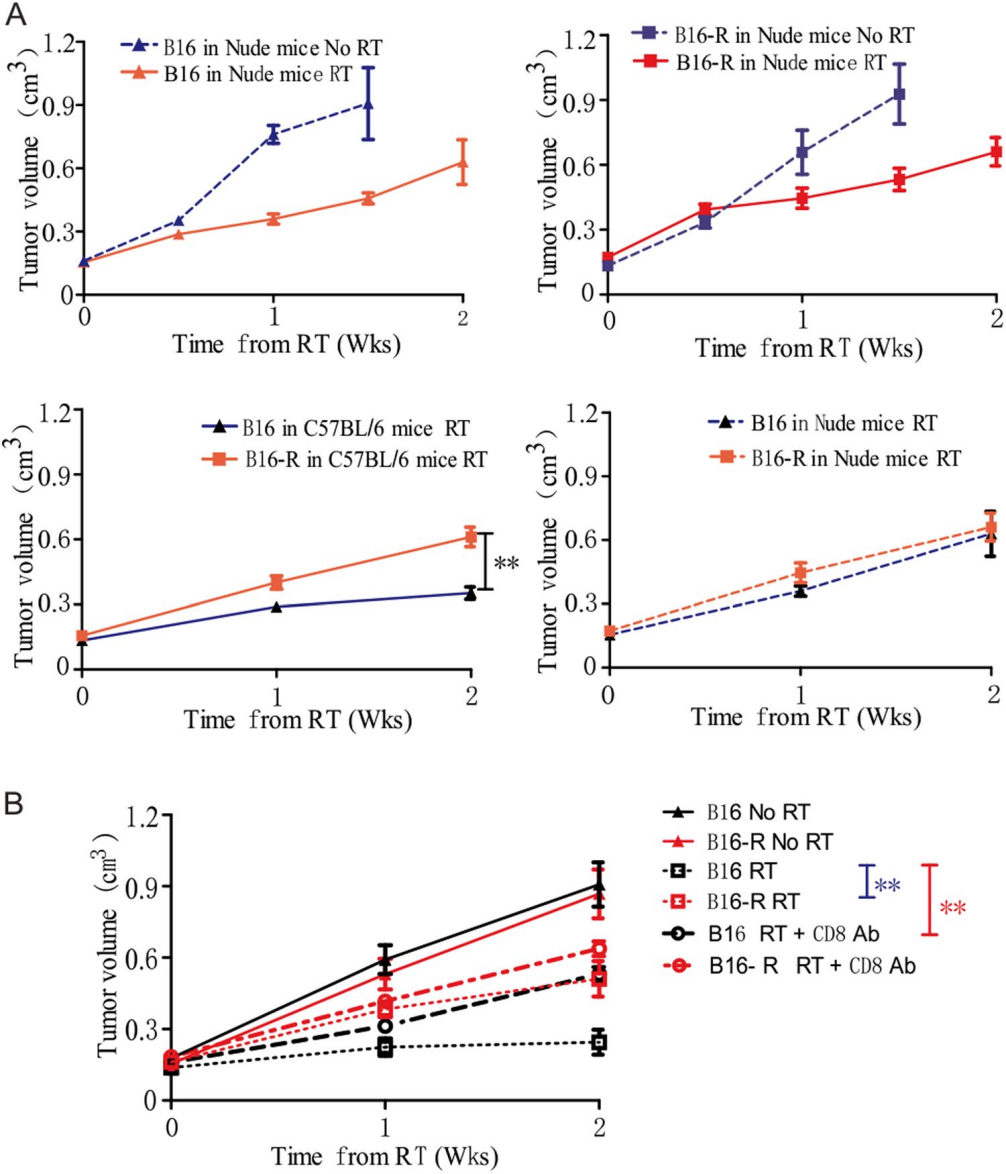
CD8^+^ T cell is associated with tumor radiosensitivity. (**A**) Nude or WT C57BL/6 mice were injected with 5 × 10^5^ B16 or B16-R cells and treated with 30 Gy. The radiation groups in nude mice showed significantly smaller tumor size compared to the no RT groups in both tumors (**p < 0.01 on day 10 from RT). The B16 tumors were smaller than the B16-R tumors in the WT C57BL/6 mice (**P < 0.01 on day 14 from RT) but not in nude mice. (**B**) Tumor growth curve for mice treated with 30 Gy and CD8 antibody. After RT plus depletion of CD8, the tumor size was larger than that after RT alone (**P < 0.01 on day 14th from RT) in the parental tumor group, but was similar in the resistant group (P > 0.05). The difference of the parental and resistant tumor size shrank after depleting CD8 compared to RT alone (from **P < 0.01 to P > 0.05 on day 14).

To determine the role of T cell infiltration in tumor radiosensitivity, tumors were implanted into athymic nude mice. A grow delay of both parental and resistant tumors in nude mice after radiotherapy was observed. When compared with the tumor volume in the nude mice, the parental tumor was about 50% smaller in the C57BL/6 mice after radiotherapy; the discrepancy of the resistant tumor volume was not so significant (Fig. 4A). The parental tumor responded well in T cell efficient mice but poor in T cell deficient mice; the difference of radiosensitivity between the parental and resistant tumor in immunocompetent mice shrank in T cell-deficient mice. To test whether CD8^+^ T cell, the major killer T cell, is essential for RT-mediated tumor reduction, we treated tumor bearing wild type C57BL/6 mice with RT in conjunction with antibody-mediated CD8^+^ T cell depletion. The therapeutic effect of RT in the parental tumor partly diminished, and the grow retard did not differ significantly between the parental and resistant tumor after depleting CD8^+^ T cell (Fig. 4B). This confirmed that most of the sensitivity of parental tumor was due to a CD8^+^ T-cell mediated immune response.

### The expression of T cell chemokines is different after irradiation in the parental and resistant tumor cells

**Figure 5:**
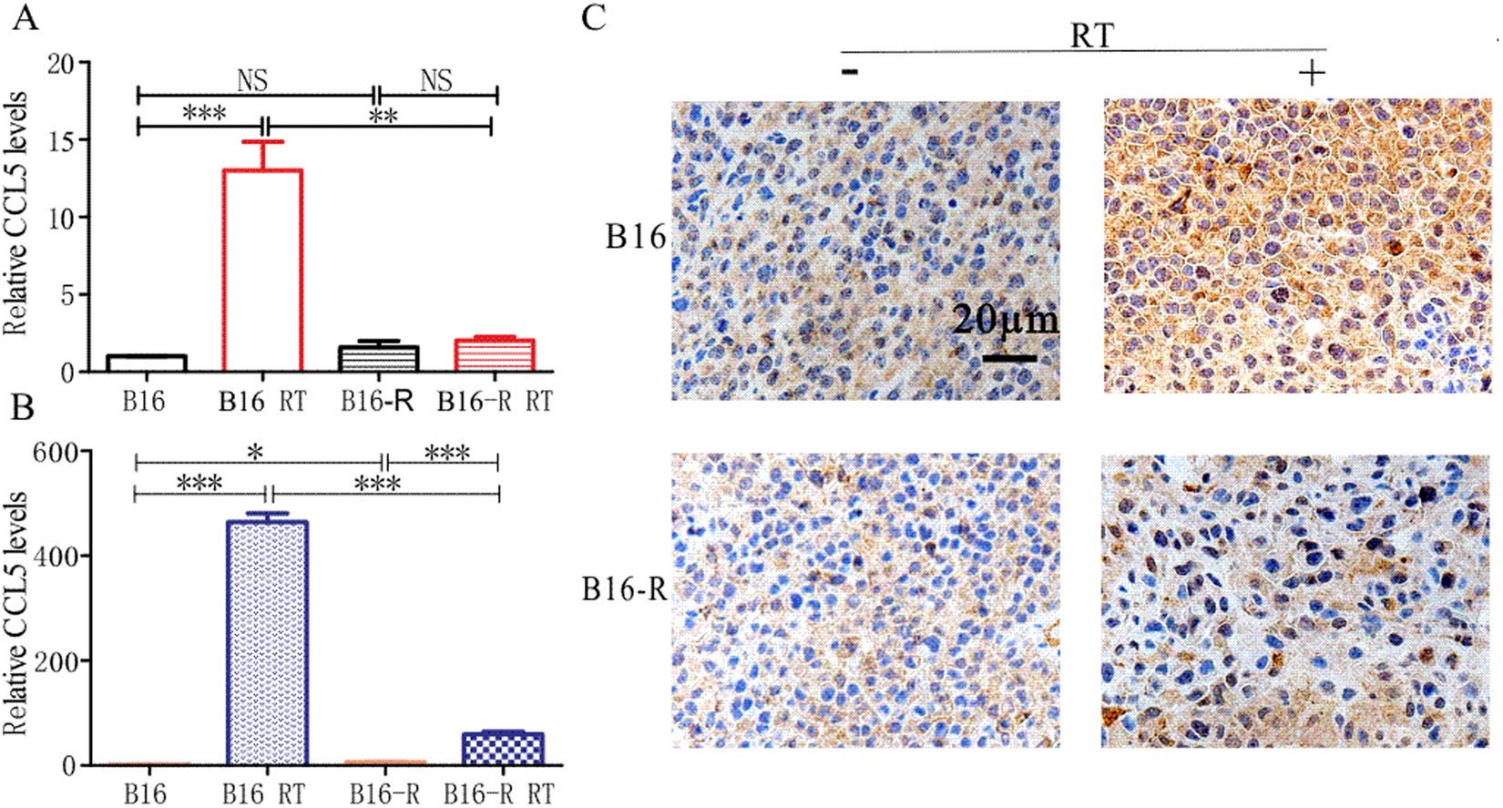
Chemokine expression is different in the parental and resistant tumor cells. After radiotherapy, the transcription of CCL5 increased significantly in the parental tumor both *in vitro* (**A**, 72 h after RT) and *in vivo* (**B**, 10 days after RT). (**C**) The expression of CCL5 was much higher in the parental tumor tissue 10 days after 30 Gy, compared to the resistant tumor (magnification × 40).

The recruitment of T cells into the tumor lesions depends on an intricate network of guiding cues and involves chemokines secreted from the tumor milieu^17,18^. Chemokines promote T cell homing, and lack of critical chemokines in a subset of melanoma may limit the migration of activated T cells^19,20^. Irradiation was reported to induce the secretion of T cell chemotactic factors by cancer cells^21–23^, so we checked their transcription. *In vitro*, the chemokines increased after radiotherapy in the parental tumor cell, and CCL2-5, CXCL9, CXCL10, CXCL16 were much higher than in the resistant cell (Figs 5A, S3). *In vivo*, CCL2-5, CXCL10 and CXCL16 also increased after radiotherapy and were with higher level in the parental tumors (Figs 5B, S3). Taken the transcription of the chemokines *in vivo* and vitro together, CCL5 had greater difference between the parental and resistant cell, we further checked its expression in the tumor tissues by IHC. The expression of CCL5 was much higher in the parental tumor cells after RT than that in the resistant tumor (Fig. 5C).

## Discussion

The underlying theory behind immune surveillance is that many tumors are eliminated by the immune system, while some cancers develop ways to escape the immune surveillance and establish a clinical apparent tumor^24^. Except the suppressive mechanisms expressed at baseline, some immune escape mechanisms manifest after treatment, tumor cells acquire counter-regulatory pathways and survive the treatment. In our model, the TME became T cell inflamed after ablative radiotherapy, but some tumor cells gained immune escape capacity through editing a less CD8 T cell infiltrated microenvironment after treatment, and became radioresistant.

The tumor response to local RT *in vivo* is not completely dependent on endogenous radiosensitivity of tumor cells. This was also reported in other studies^21,25,26^. Twyman *et al*.^25^ gained a radioresistant cell line from tumor relapsed after radiation and anti-CTLA4 treatment, and resistance was confirmed *in vivo* but had similar clonogenic survival *in vitro*. In the study from Aguilera *et al*.^26^, different mammary tumor cell clones that responded differently to the same dose *in vivo* had similar radiosensitivity in cell culture. Recent studies have demonstrated that local ablative radiation of established tumors can lead to increased T cell priming and T cell dependent tumor regression^3,11,12,27,28^. RT dramatically increases cytotoxic T-cell infiltration, and the therapeutic effect of irradiation markedly attenuates in nude mice or after depleting CD8^+^ T cells. In radioresistant tumor, the density of CD8 T cell did not change a lot after radiotherapy, and responded similarly whether in a wild-type or immune-compromised host. The disparate regrowth rate between the parental and resistant tumor downsized in T cell deficient mice, which also implicated that much of the difference of radiosensitivity in immunocompetent mice is due to the aggregation of T cells in sensitive tumor. More CD8^+^ T cell infiltration is not only a manifestation of better response, more importantly, it results in better tumor shrinkage. Through inhibiting the aggregation of CD8^+^ T cells after radiotherapy, tumors become radioresistant.

The ability of RT to invoke a systemic immune response and the immune system’s role in the local effect of RT underscore the potential clinical utility of RT and immunotherapy, but many patients do not respond to this combination^29^. In this study, patients did not have uniform change in the immunological microenvironment after radiotherapy, some of the patients had robust CD8^+^ T cell infiltration, and some only had moderate increase, which was in accordance with other studies^30–32^. This may partly explain why the combination of RT and immunotherapy is useless in some patients, as sufficient T cell infiltration in tumor tissues is a prerequisite for response to checkpoint inhibitors^33^. For these patients, overcoming the non-T cell-inflamed TME after radiotherapy is a big problem.

The trafficking of T cells is a complex process and affected by many factors, one of which is the expression of chemokines. The chemokines increased after radiotherapy in the parental tumors, further functional study is in need to support its role in T cell aggregation. Through some unknown mechanisms, the increase of chemokine was offset in the resistant tumors. The expression of chemokines is controlled by some transcription factors such as NF-κB^34^, STAT^35^, Axl^26^ *et al*. Tumors that contained a T cell infiltrate were reported to show evidence of a transcriptional signature known to be induced by type I interferon^36^, and in the analysis of the transcriptional cascade induced by irradiation, Purbey PK *et al*.^37^ demonstrated that most of the late response genes corresponded to interferon-stimulated genes. Interferon induced the production of cytokines^38^. Gain-of-function mutations in β-catenin was reported to be more frequently observed in the non-T-cell-infiltrated melanoma tissues, and the tumor cell-β-catenin activation was sufficient to exclude T cells infiltrates *in vivo*^39^. In the study from Aguilera TA *et al*.^26^, the expression of Axl was different in the resistant and wild type tumor cell sub-clone, and Axl knockout resulted in higher expression of MHC I and STAT1, and chemokines responsible for myeloid cells recruitment. It was unknown whether these reported genes correlated to less CD8 T cell recruitment after radiotherapy in the resistant tumors. Further study is going on in order to clarify this problem in our group.

Besides chemokines, there are many other factors that may interfere the attraction of T cells, such as tumor mutation burden and the antigen presentation capacity of dendritic cells, *et al*. Details of the control of T cell infiltration after radiation, and the search of inherent genes or signal pathways in the tumor cells responsible for modifying T cell infiltration before and after radiation, may help to detect new candidate targets to improve immune potentiation and radiosensitivity.

We observed that there was more MDSC infiltration at baseline, which decreased after radiotherapy, and there was a significant change of the percentage of CD11b^+^Ly6G^+^ and CD11b^-^Ly6C^+^ in the parental tumor. Considering the suppressive role of MDSC, it is possible that MDSC is an important immunosuppressive factor in the parental tumor, and aside from the more CD8^+^ T cells infiltration, the decrease of MDSC and the change of the subtypes after radiotherapy may be another important mechanism for radiosensitivity. Future study is needed to explore this hypothesis.

In conclusion, this study demonstrates a novel mechanism of radioresistance, that is, tumor cells inhibit the infiltration of CD8^+^ T cell after radiotherapy and become radioresistant, the infiltration of CD8^+^ T cell after radiotherapy is an important determinant of radiosensitivity. Ways to increase the infiltrated T cells after radiotherapy may be a potential candidate for improving radiation response.

## Methods

### Cell lines

The melanoma cell line B16 was obtained from the American Type Culture Collection (ATCC, USA). In order to gain the radio-resistant cell line B16-R, 6–8 weeks old C57BL/6 mice were obtained, and 5 × 10^5^ B16 tumor cells suspended in 100 ul PBS were inoculated orthotopicly in the right inguinal region of each mouse (as follows). 45 Gy in three fractions was given to the tumor lesion one and the other day. Two to three weeks later those tumors responded poor to RT were excised and digested into single cell, and cultured in RPIM1640 complete medium. When the cells were in increased logarithmic phase, they were collected and re-inoculated into the mice. The above procedure was repeated, and the radiation response *in vivo* was compared with the parental tumor, after 225 Gy we gained a resistant tumor cell which showed faster tumor regrowth after RT in C57BL/6 mice.

### Tumor treatments

5 × 10^5^ B16 or B16-R tumor cells were inoculated orthotopicly in the C57BL/6 or nude mice. RT of tumors was typically initiated between 100 mm^3^ and 200 mm^3^, 30 Gy in 2 fractions on one and the other day. This radiation dose and fraction size was selected after pilot experiments showed that this combination was optimal for primary tumor regression and control. Animals were observed for primary tumor control and survival. Volume was calculated as 0.52 × L × W^2^ every 2-3 days. For irradiation treatments, animals were anesthetized with 2% phenobarbital sodium, and restrained in a custom-designed irradiation jig, ensuring that only the tumor was irradiated. For irradiation treatments, an X-RAD 320 irradiation cabinet (Precision X-Ray, East Haven, CT) was utilized at 320 kV and 160 mA, at a target-source distance of 20 cm and a dose rate of 3.4 Gy/min. Animal protocols in the study were approved by the Institutional Animal Care and Use Committee of Renji Hospital, School of Medicine, Shanghai JiaoTong University.

For blocking antibody experiments, a total of 200 μg/mouse anti-CD8 antibody (clone 53.6.7, Sungenebiotech, China) was administered on days 0, 4, 8 and 12 after RT intraperitoneally. Each group included 7–9 mice.

### Cell line irradiation

B16 and B16-R cells were irradiated by an X-RAD 160 irradiation cabinet (Precision X-Ray, East Haven, CT) with 160 kV and 14.46 mA, at a target-source distance of 60 cm and a dose rate of 1.0 Gy/min. Cells were further incubated up to different time points and harvested for the subsequent experiments.

### Clonogenic assay

1 × 100 to 2 × 10^4^ cells (B16 or B16-R)/ well were plated in 6-well plate, and irradiated at different doses ranging from 0 to 8 Gy, and incubated for 10–14 days. Colonies consisting of 50 cells or more were counted and the surviving fraction was determined. All survival curves represented at least three independent experiments.

### Evaluation of death by Annexin and propidium iodide (PI) staining

48 h after 10 Gy, cells were harvested and incubated according to the instruction (Annexin V-FITC kit; B & D). Annexin V-positive cells or PI-positive cells were considered to be dead. Cells without irradiation were used as normal control. Three independent experiments were performed.

### Western blot

Cells were collected and lysed, the protein was separated using 12% polyacrylamide gel, and transferred to polyvinyl difluoride membranes. After blocked with 5% bovine serum albumin and incubated with specific primary antibodies (anti-β-actin monoclonal (Santa Cruz Biotechnology, USA) or anti-γ H2AX monoclonal (Cell Signaling Technology, USA)), the membranes were incubated with anti-mouse or anti-rabbit IgG secondary antibody (Santa Cruz Biotechnology), then the blots were developed using an ECL western blotting detection reagent (GE Healthcare Bio-Sciences Corp., UK) and Luminoimage Analyzer LAS-3000 (Fuji, Japan).

### Cell preparation and Flow cytometric analysis

Mice were sacrificed 14 days from irradiation or not. Tumor tissues were resected and weighed, then digested into single cell. Mononuclear cells in the TME were enriched using Lympholite M gradients (CedarlaneLaborato-ries). Two independent experiments of 7–9 mice per group were done.

The collected cells were blocked with Fc blocker, incubated with anti-CD45 antibody (FITC, clone 30-F11) and 7AAD (cat # 640930). CD45^+^7AAD^−^ were gated as TIL for further analysis (see Fig. S4). Additional antibodies were used to detect CD4^+^ T cell (anti-CD3-PE, clone 17A2; anti-CD4-APC, clone GK1.5), CD8^+^ and CD3^+^ T cell (anti-CD3-PE, clone 17A2; anti-CD8-APC, clone 53-6.7; anti-CD44-BV421, clone BJ18; anti-62L-AF700, clone DREG56), MDSCs (anti-Gr-1-PE, clone RB6-8C5; anti-CD11b-APC, clone M1/70) or (anti-CD11b-APC, clone M1/70; anti-Ly6G-PE, clone 1A8; anti-Ly6C-BV421, clone HK1.4), B cells and dendritic cells (anti-MHCII-PE, clone 15-5-5; anti-CD11c-APC, clone N418). The antibodies were purchased from Biolegend or Ebioscience, and used according to the instructions. Data was analyzed by flow cytometry using a Becton-Dickinson FACS flow cytometer with Accuri CFlow software (BD, USA) and FlowJo Software (Ashland, USA).

### Quantitative real time-polymerase chain reactions (qRT-PCR)

Xenograft tumor pieces were collected by resection, and stored in the liquid nitrogen (preferred) or −80 °C until processing. Total RNA was isolated using TRIzol reagent (Invitrogen). RNA was reverse-transcribed by Revert Aid First Strand cDNA Synthesis Kit (Thermo Scientific) according to the manufacturer’s instructions. Quantitative real-time PCR was performed using SYBR Green PCR real-time PCR Master MIX (Toyobo). The expression level of GAPDH was used as an internal control for normalization. Primers were shown in Table S1.

### Histopathology and TRG

During 2015 to 2016, 52 patients who received neoadjuvant chemoradiotherapy for rectal cancer in our department were collected. 46–50.4 Gy was given to the tumor with 1.8 to 2 Gy per fraction, concurrent with capecitabine. Surgical treatment was carried out 6–8 weeks after treatment. Among them 32 patients had diagnostic biopsy before treatment in our hospital, the biopsy specimens were also retrieved. All procedures were conducted in accordance with the Helsinki declaration, and with approval from the Ethics Committee of RenJi Hospital. Written informed consent was obtained from all participants.

HE staining and immunohistochemistry (IHC) of CD8 (CD8 antibody, 1:50, Proteintech) were performed on 4-μm serial sections on all samples.

Tumor response was evaluated using the TRG system as described^15,16^. CD8^+^ T cell was scored on 3–5 high-power fields in the tumor areas of highest density of CD8-positive cells by two investigators who were blind to the TRG, and the average count was recorded.

For animal model, melanoma tissues from mice at indicated time were resected, fixed in formalin then embedded in paraffin, and anti-CCL5 antibody (1:100, Novus) was used to detect the CCL5 by IHC.

### Statistical analysis

Statistical analysis was performed using SPSS version 21 software. The comparisons of categorical variables were evaluated using the χ^2^ or Fisher’s exact tests. Significant differences were determined using a paired t test in the same group and the Mann-Whitney U test across different groups. *p < 0.05, **p < 0.005, and ***p < 0.001.

All data generated or analyzed during this study are included in this published article and its Supplementary Information files.

## Electronic supplementary material

Dataset1

## References

[1] Derer A, Frey B, Fietkau R, Gaipl US (2016). Immune-modulating properties of ionizing radiation: rationale for the treatment of cancer by combination radiotherapy and immune checkpoint inhibitors. Cancer Immunol Immunother.

[2] Gerweck LE, Vijayappa S, Kurimasa A, Ogawa K, Chen DJ (2006). Tumor cell radiosensitivity is a major determinant of tumor response to radiation. Cancer Res.

[3] Lee Y (2009). Therapeutic effects of ablative radiation on local tumor require CD8^+^ T cells: changing strategies for cancer treatment. Blood.

[4] Apetoh L (2007). Toll-like receptor 4-, dependent contribution of the immune system to anticancer chemotherapy and radiotherapy. Nat Med.

[5] Vanpouille-Box C (2015). TGFβ is a master regulator of radiation therapy-induced antitumor immunity. Cancer Res.

[6] Kroemer G, Galluzzi L, Kepp O, Zitvogel L (2013). Immunogenic cell death in cancer therapy. Annu Rev Immunol.

[7] Kalbasi A, June CH, Haas N, Vapiwala N (2013). Radiation and immunotherapy: a synergistic combination. The journal of clinical investigation.

[8] Jeong H, Bok S, Hong BJ, Choi HS, Ahn GO (2016). Radiation-induced immune responses: mechanisms and therapeutic perspectives. Blood research.

[9] Formenti SC, Demaria S (2013). Combining radiotherapy and cancer immunotherapy: a paradigm shift. J Natl Cancer Inst.

[10] Whiteside TL, Demaria S, Rodriguez-Ruiz ME, Zarour HM, Melero I (2016). Emerging opportunities and challenges in cancer immunotherapy. Clin cancer res.

[11] Takeshima T (2010). Local radiation therapy inhibits tumor growth through the generation of tumor-specific CTL: its potentiation by combination with Th1 cell therapy. Cancer Res.

[12] Gupta A (2012). Radiotherapy promotes tumor-specific effector CD8^+^ T cells via dendritic cell activation. J Immunol.

[13] Takeshima T (2016). Key role for neutrophils in radiation-induced antitumor immune responses: Potentiation with G-CSF. Proc Natl Acad Sci USA.

[14] Liang H (2017). Host STING-dependent MDSC mobilization drives extrinsic radiation resistance. Nat Commun.

[15] Ryan R (2005). Pathological response following long-course neoadjuvant chemoradiotherapy for locally advanced rectal cancer. Histopathology.

[16] Bozzetti F, Andreola S, Bertario L (1998). Pathological features of rectal cancer after preoperative radiochemotherapy. Int J Colorectal Dis.

[17] Sackstein R, Schatton T, Barthel SR (2017). T-lymphocyte homing: an underappreciated yet critical hurdle for successful cancer immunotherapy. Lab Invest.

[18] Slaney CY, Kershaw MH, Darcy PK (2014). Trafficking of T cells into tumors. Cancer Res.

[19] Hong M (2011). Chemotherapy Induces Intratumoral Expression of Chemokines in Cutaneous Melanoma, Favoring T-cell Infiltration and Tumor Control. Cancer Res.

[20] Harlin H (2009). Chemokine Expression in Melanoma Metastases Associated with CD8^+^ T-Cell Recruitment. Cancer Res.

[21] Matsumura S (2008). Radiation-induced CXCL16 release by breast cancer cells attracts effector T Cells. The Journal of Immunology.

[22] Demaria S, Bhardwaj N, McBride WH, Formenti SC (2005). Combining radiotherapy and immunotherapy: a revived partnership. Int. J. Radiat. Oncol. Biol. Phys..

[23] Demaria S, Formenti SC (2007). Sensors of ionizing radiation effects on the immunological microenvironment of cancer. Int. J. Radiat. Biol..

[24] Pardoll D (2015). Cancer and the immune system: Basic concepts and targets for intervention. Semin Oncol.

[25] Twyman-Saint VC (2015). Radiation and dual checkpoint blockade activate non-redundant immune mechanisms in cancer. Nature.

[26] Aguilera TA (2016). Reprogramming the immunological microenvironment through radiation and targeting Axl. Nat Commun.

[27] Burnette BC (2011). The efficacy of radiotherapy relies upon induction of type i interferon-dependent innate and adaptive immunity. Cancer Res.

[28] Liang H (2013). Radiation-Induced Equilibrium Is a Balance between Tumor Cell Proliferation and T Cell–Mediated Killing. J Immunol.

[29] Salama AK, Postow MA, Salama JK (2016). Irradiation and immunotherapy: from concepts to the clinic. Cancer.

[30] Shinto E (2014). CD8+ and FOXP3+ tumor-infiltrating T cells before and after chemoradiotherapy for rectal cancer. Ann Surg Oncol.

[31] Teng, F. *et al*. Tumor infiltrating lymphocytes (TILs) before and after neoadjuvant chemoradiotherapy and its clinical utility for rectal cancer. *Am J Cancer Res* **5**(6), 2064–74, eCollection (2015). PMC4529625 26269765

[32] Homma Y (2014). Immunological impact of neoadjuvant chemoradiotherapy in patients with borderline resectable pancreatic ductal adenocarcinoma. Ann Surg Oncol.

[33] Tang H (2016). Facilitating T Cell Infiltration in Tumor Microenvironment Overcomes Resistance to PD-L1 Blockade. Cancer Cell.

[34] Karin M, Greten FR (2005). NF-kappaB: linking inflammation and immunity to cancer development and progression. Nat Rev Immunol.

[35] Yu H, Pardoll D, Jove R (2009). STATs in cancer inflammation and immunity: a leading role for STAT3. Nat Rev Cancer.

[36] Gajewski TF (2015). The next hurdle in cancer immunotherapy: Overcoming the non-T cell-inflamed tumor microenvironment. Semin oncol.

[37] Purbey PK (2017). Defined Sensing Mechanisms and Signaling Pathways Contribute to the Global Inflammatory Gene Expression Output Elicited by Ionizing Radiation. Immunity.

[38] Borden EC (2007). Interferons at age 50: past, current and future impact on biomedicine. Nat Rev Drug Discov.

[39] Spranger S, Bao R, Gajewski TF (2015). Melanoma-intrinsic β-catenin signalling prevents anti-tumour immunity. Nature.

