# Supplementary material for: Inhibiting the CD8+ T cell infiltration in the tumor microenvironment after radiotherapy is an important mechanism of radioresistance
Source: Sci Rep. 2018 Aug 9;8:11934. doi: 10.1038/s41598-018-30417-6 (PMC6085329; doi:10.1038/s41598-018-30417-6)

Inhibiting the $CD8^+$ T cell infiltration in the tumor microenvironment after radiotherapy is an important mechanism of radioresistance

Hai-yan Chen, Lei Xu, Lin-feng Li, Xiao-xing Liu, Jian-xin Gao, Yong-rui Bai

Supplementary dataset 1

Tab. S1. Primers used in the qRT−PCR.

Gene	Forward primer	Backward primer
GAPDH	TGGCCTTCCGTGTTCCTAC	GAGTTGCTGTTGAAGTCGCA
CCL2	TTAAAAACCTGGATCGGAACCAA	GCATTAGCTTCAGATTTACGGGT
CCL3	TTCTCTGTACCATGACACTCTGC	CGTGGAATCTTCCGGCTGTAG
CCL4	TTCCTGCTGTTTCTCTTACACCT	CTGTCTGCCTCTTTTGGTCAG
CCL5	GCTGCTTTGCCTACCTCTCC	TCGAGTGACAAACACGACTGC
CXCL9	GGAGTTCGAGGAACCCTAGTG	GGGATTTGTAGTGGATCGTGC
CXCL10	CCAAGTGCTGCCGTCATTTTC	GGCTCGCAGGGATGATTTCAA
CXCL11	GGCTTCCTTATGTTCAAACAGGG	GCCGTTACTCGGGTAAATTACA
CXCL16	GTGGCTTCTTGGGGGTATGG	TCTTAGTGGGTGGCGTCATTA

Supplementary dataset 2

Figure-S1. Full-length blots/gels.

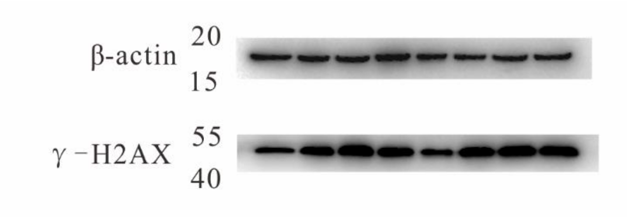

Supplementary dataset 3

Figure-S2. The change of MDSC in the tumor microenvironment after radiotherapy. After radiotherapy, there was a higher group of CD11b^{-}Ly6C^{+} and less CD11b^{+}Ly6G^{+} cells in the tumor infiltrating leucocytes of the parental tumor.

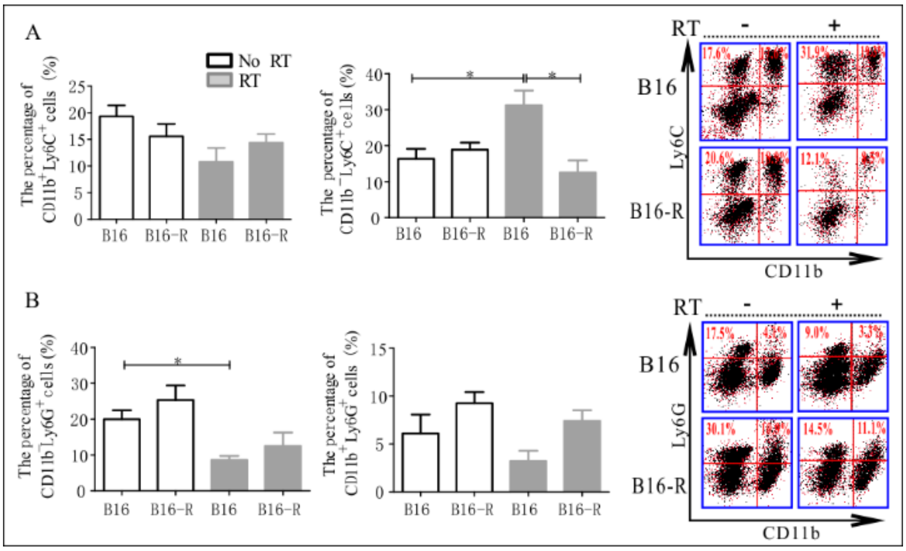

Supplementary dataset 4

Figure-S3. The transcription of T cell chemokines. The comparison of transcription of the chemokines before and after irradiation in both cells in vitro (A) and in vivo (B).

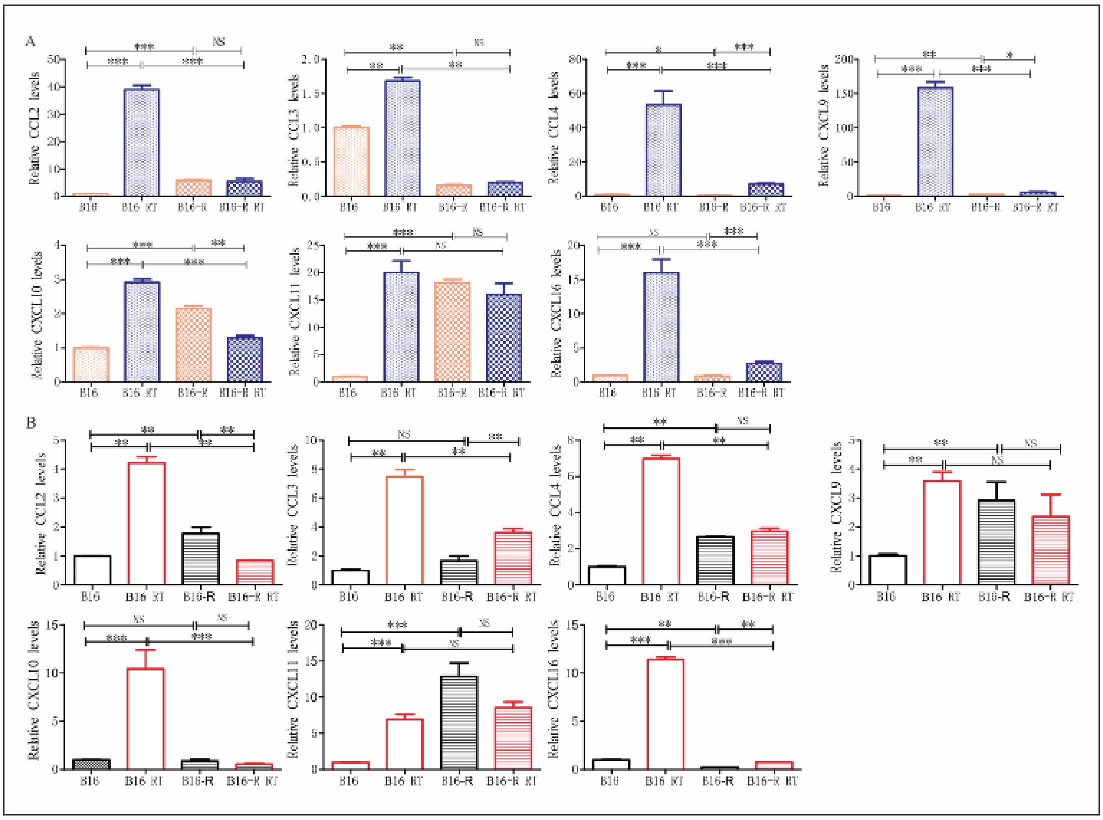

Supplementary dataset 5

Figure-S4. Flow cytometry and gating of tumor infiltrating leucocytes from digested tumor specimens. CD45^{+} 7AAD^{-} cells were gated as tumor infiltrating leucocytes for further analyzed.

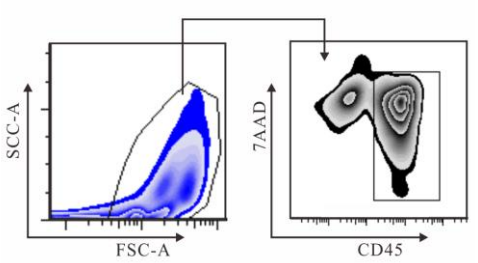

Supplement: Supplementary file 1 — Dataset1 [file 41598_2018_30417_MOESM1_ESM.pdf]
